# The miRNA 361-3p, a Regulator of GZMB and TNF Is Associated With Therapeutic Failure and Longer Time Healing of Cutaneous Leishmaniasis Caused by *L. (viannia) braziliensis*

**DOI:** 10.3389/fimmu.2018.02621

**Published:** 2018-11-14

**Authors:** Tainã S. Lago, Juliana Almeida Silva, Ednaldo L. Lago, Edgar M. Carvalho, Dalila L. Zanette, Léa Cristina Castellucci

**Affiliations:** ^1^Serviço de Imunologia, Universidade Federal da Bahia, Salvador, Brazil; ^2^Laboratório de Investigação em Genética e Hematologia Tanslacional do Instituto Gonçalo Moniz–Fiocruz-Ba, Salvador, Brazil; ^3^Laboratório de Pesquisa Clínica (LAPEC) do Instituto Gonçalo Moniz–Fiocruz-Ba, Salvador, Brazil; ^4^Instituto Nacional de Ciência e Tecnologia em Doenças Tropicais (INCT-DT), Salvador, Brazil; ^5^Programa de Pós-graduação em Ciências da Saúde, Universidade Federal da Bahia, Salvador, Brazil

**Keywords:** miRNAs, American cutaneous leishmaniasis, gene expression, tissue damage, immunopathogenesis

## Abstract

*L. (viannia) braziliensis* infection causes American Tegumentary Leishmaniasis (ATL), with prolonged time to healing lesions. The potent inflammatory response developed by the host is important to control the parasite burden and infection however an unbalanced immunity may cooperate to the tissue damage observed. The range of mechanisms underlying the pathological responses associated with ATL still needs to be better understood. That includes epigenetic regulation by non-coding MicroRNAs (miRNAs), non-coding sequences around 22 nucleotides that act as post-transcriptional regulators of RNAs encoding proteins. The miRNAs have been associated with diverse parasitic diseases, including leishmaniasis. Here we evaluated miRNAs that targeted genes expressed in cutaneous leishmaniasis lesions (CL) by comparing its expression in both CL and normal skin obtained from the same individual. In addition, we evaluated if the miRNAs expression would be correlated with clinical parameters such as therapeutic failure, healing time as well as lesion size. The miR-361-3p and miR-140-3p were significantly more expressed in CL lesions compared to normal skin samples (*p* = 0.0001 and *p* < 0.0001, respectively). In addition, the miR-361-3p was correlated with both, therapeutic failure and healing time of disease (*r* = 0.6, *p* = 0.003 and *r* = 0.5, *p* = 0.007, respectively). In addition, complementary analysis shown that miR-361-3p is able to identify with good sensitivity (81.2%) and specificity (100%) patients who tend to fail initial treatment with pentavalent antimonial (Sbv). Finally, the survival analysis considering “cure” as the endpoint showed that the higher the expression of miR-361-3p, the longer the healing time of CL. Overall, our data suggest the potential of miR-361-3p as a prognostic biomarker in CL caused by *L. braziliensis*.

## Introduction

American tegumentar leishmaniasis (ATL) is a complex, multifactorial disease that results from environmental factors such as parasite polymorphisms, phlebotomine sand fly components, as well as the host's immune and genetic background (1). This disease extends from Mexico to Argentina (2), and is caused by various species of the *Leishmania braziliensis* and *Leishmania mexicana* complexes of parasites (3). *Leishmania (Viannia) braziliensis* is responsible for the majority of ATL cases in South America, causing localized cutaneous (CL), mucosal leishmaniasis (ML), and disseminated leishmaniasis (DL) in Northeast Brazil. Patients with CL and ML typically present a potent inflammatory response characterized by the production of high levels of IFN-γ and tumor necrosis factor alpha (TNF) (4–6). Although important to control infection, the exacerbated inflammation leads to the tissue damage observed in cutaneous and mucosal ulcers. This process is determined by the selection and activity of macrophages, NK cells, TCD4+, and TCD8+ cells. Studies have shown that CD8+ T cells are associated with tissue damage in cutaneous leishmaniasis due to a destructive inflammatory response and a high number of cytotoxic cells in blood and tissues (7–10). In addition, data have shown that in *L. braziliensis* infected patients, there is inflammasome activation, generating IL-1β, which increases the inflammation, stimulating the production of chemokines and also matrix metalloproteinases, that degrade the extracellular matrix leading to tissue damage (9). DL compared to CL has been associated with lower IFN-γ and TNF production, higher IL-5 production, and anti-Leishmania antibodies, with variable T cell response among affected individuals (11). As a whole, this complex disease depends on both the extent of parasite elimination and the relative induction of potentially immunopathologic responses (7). In this context, we believe that host-parasite interaction hides key mechanisms of disease outcome and this interface is finely regulated by epigenetic mechanisms. Broadly speaking, epigenetics refers to stimuli-triggered changes in gene expression due to processes that arise independent of changes in the underlying DNA sequence. Some of these processes have been elucidated and include DNA methylation (12), histone modifications and chromatin-remodeling proteins (13), and DNA silencing by non-coding RNAs, including miRNAs (14). The microRNAs are non-coding RNAs consisting of a sequence of about 22 nucleotides, which act as post-transcriptional regulators by interfering in processing and stability of RNA coding proteins. Since their identification, miRNAs have been associated to numerous biological and pathological processes, including several inflammatory and infectious diseases (15–19). Regarding leishmaniasis although there is now an increasing body of evidence underpinning alterations of these small RNAs in different models (20–23), there is a paucity of studies performed with human samples. As examples, data showed changes in the expression of miRNAs in human macrophages infected by *L. major*, with some of these miRNAs involved in the activation of monocytes through TLR signaling (24). In addition, there was a species-specific global downregulation in miRNA expression among host cells following infection with *L. major* and *L. donovani*, which showed enrichment of MAP kinase, JAK-STAT, and TGF-β signaling pathways after *in silico*- predicted targets analysis (25). Finally, in a recent study, the miR-193b, miR-671, and *TREM1* were correlated with faster wound healing in patients infected by *L. braziliensis* (26). All together these data support the idea that the host cell undergoes selection and specific miRNA regulation after infection with *Leishmania* parasites. However, it is necessary to add to the literature data on how these molecules are expressed in specimens of patients. In this study we used a previously published transcriptional analysis comparing CL lesion samples and normal skin of patients infected *L. braziliensis* (GEO accession number GSE55664), which showed that genes associated with inflammatory cell recruitment (*CXCL9, CXCL10*, and *CCL8*) and cytotoxicity (*GZMA, GZMB*, and *GLYN*) were highly expressed in *L. braziliensis* lesions. In contrast, the most down-regulated genes were associated with maintenance of the epidermal barrier function (8). Based on this previous study, we aimed to identify the miRNAs that could act as regulators of differentially genes found in CL lesions and thus, be potential markers of immune response and tissue damage, as well as modifiers of clinical parameters such as disease severity, response to treatment and healing time.

## Materials and methods

### The endemic site

The study was conducted in the rural area of Corte de Pedra, Bahia, Brazil, where ATL caused by *L. braziliensis* is endemic. The area of Corte de Pedra is characterized by isolated sites of Atlantic forest with agricultural activities providing the main source of income for the majority of its inhabitants (27). The occupational and domestic habits of these individuals, which involve work on farms and homes built in clearings in the woods, have increased the population exposure to *L. braziliensis* infection. Between the years 2012 and 2017, 6,597 cases of ATL were reported in the region of Corte de Pedra. As other endemic *foci*, the cure rates of antimonial pentavalent (Sbv) declined from 90 to 50% in the last 30 years (28–30).

### Patient recruitment and clinical data

Twenty four patients with CL, enrolled after diagnosis and prior treatment, of both sexes and aged between 18 and 60 years, were recruited at the health clinic of Corte de Pedra-BA. Patients who had other clinical forms of the disease, pregnant and carriers of systemic diseases were excluded. CL was defined by the presence of one or a few ulcerative cutaneous lesions with raised borders and no evidence of mucosal involvement. All patients had clinical diagnosis confirmed by DNA detection of the parasite by PCR in biopsy specimens. Demographic features, illness duration, number of lesions, location, and size of the ulcers were recorded. All patients were treated with antimoniate of meglumine (Glucantime, Sanofi/Avetis), as recommended by the Brazilian Minister of Health. The Glucantime (Sanofi/Aventis) was administration by intravenous route daily in a concentration of 20 mg/Kg/day for 20 days. Cure was defined as a complete healing of ulcers in the absence of raised borders on day 90 and failure by persistence of active ulcer or healing of the lesion but with raised edges. All these data were taken into account in our analysis, in an attempt to identify a correlation between these data and the miRNA expresion. Finally, we classified the patients in two groups according to treatment response to Sbv. We defined as “responders,” those who cured their lesion with a single course of Sbv on day 90; while “refractories” were those who needed two or more courses of Sbv or other alternative drug and took more than 90 days to heal their lesions. This stratification was used to perform correlation analysis regarding therapy failure and healing time.

### Sample collection and RNA extraction

At the clinical diagnosis and prior to treatment, two tissue specimens were obtained from each patient: a normal skin biopsy was took from a distant site of the CL lesion, whereas a CL biopsy was collected from the border of the lesion. These biopsies were collected by a physician, using a 4 mm punch and immediately preserved in RNA Later Solution (Ambion), until further RNA isolation. The tissue was intensely macerated using the TissueRuptor (Qiagen) followed by TRIzol RNA isolation, according to manufacturer's instructions. The isolated RNA was resuspended in 20 μl of RNase-free water and concentration determined by optical density measurements (260 and 280 nm) using Nanodrop^®^. Samples were stored at −70°C until use.

### cDNA synthesis and evaluation of miRNA expression by quantitative RT-PCR (qPCR)

By filtering information from the public databases TargetScan Human Prediction Tool version 7.0 and miRBase (release 22) we choose candidate miRNAs that were indicated as regulators of the differentially expressed genes described by Novais et al. (8). Reverse transcription and complementary DNA (cDNA) synthesis were performed using the commercially available TaqMan^®^ Advanced miRNA cDNA Synthesis Kit (Thermo Fisher Scientific), following the manufacturer's instructions. The expression of candidate miRNAs in skin biopsies of patients with CL was evaluated by the real-time quantitative PCR (qPCR) technique using TaqMan^Ⓡ^ Advanced miRNA assays (Thermo Fisher Scientific) through the 7500 Real Time PCR System (Applied Biosystems^Ⓡ^), according to the protocol provided by the company. All reactions were performed in duplicates and levels of miRNA target expression were normalized to the corresponding endogenous hsa-miR-24-3p (31). The amplification cycling consisted of two consecutive holds of 5 min at 50°C and 10 min at 95°C, followed by 40 cycles of 15 s at 95°C and 1 min at 60°C. The expression values were obtained by the comparative delta (Δ) Ct method.

### Statistical analysis

The miRNAs data were represented in units of relative expression between comparison groups. Association tests were performed using the non-parametric Mann-Whitney. Correlation analysis between the miRNAs and clinical parameters was made by the Spearman correlation test. A survival analysis was estimated by the Kaplan-meier method with the aim of differentiating the healing time according to the miRNAs expression values. In this case, we calculated the median expression values (considering all patients) and defined that values below 7.0 (median) would be classified as low expression and values above 7.0, high expression. In addition, A ROC curve was made to predict who might cure or fail the Sbv treatment according to the miRNAs expression values. A *p* < 0.05 was considered statistically significant. For all analysis we used the GraphPad Prism 5 software.

### Ethical statement

This study adhered to the Declaration of Helsinki and was approved by the Hospital Universitário Professor Edgard Santos Ethics Committee at Universidade Federal da Bahia (CEP 22/2012) and the Brazilian National Ethical Committee (CONEP: 1258513.1.000.5537). A written informed consent was obtained from every individual who accepted to participate in this study.

## Results

### Clinical and epidemiological characteristics of the sample

Twenty-four patients with CL were recruited at the diagnosis in the Health Center of Corte de Pedra, Brazil. The clinical and demographic of these patients who cure or failure to antimony therapy are detailed on Table 1. Patients who failed were younger than those who cured. There was no difference between the groups regarding the gender, duration of the illness, number of lesions, size of the greater lesion and percentage of ulcers in the inferior limb. As expected the healing time in those who cured were shorter than in those who failed.

**Table 1:** Clinical and demographical characteristics of the CL patients.

**Demographic and clinical features**	**Treatment outcome fail**		
	**Cure (*N* = 20)**	**(*N* = 4)**	***P*-value**
Age, mean, (SD)	32 ± 9.7	22 ± 3.9	0.037
Gender, % Male Illness duration, median, (IQR) Number of lesions, median, (IQR) Size of the major lesion, median, mm2 (IQR) Percentage of lesions in the inferior limbs Healing time, median (IQR)	15/20 (75) 81.5 (65–120) 1.5 (1–2) 163.5 (84–537.5) 50 50 (36.2–59)	3/4 (60) 160 (137.5–178) 1 (1–2.5) 42 (25–301) 50 120 (120–143)	0.596 0.008 0.765 0.071 1.000 0.001

*SD, Standard deviation; IQR, Interquartile Range*.

### Evaluation of the miRNAs expression in normal and CL skin biopsies of patients infected by *L. braziliensis*

**Figure 1:**
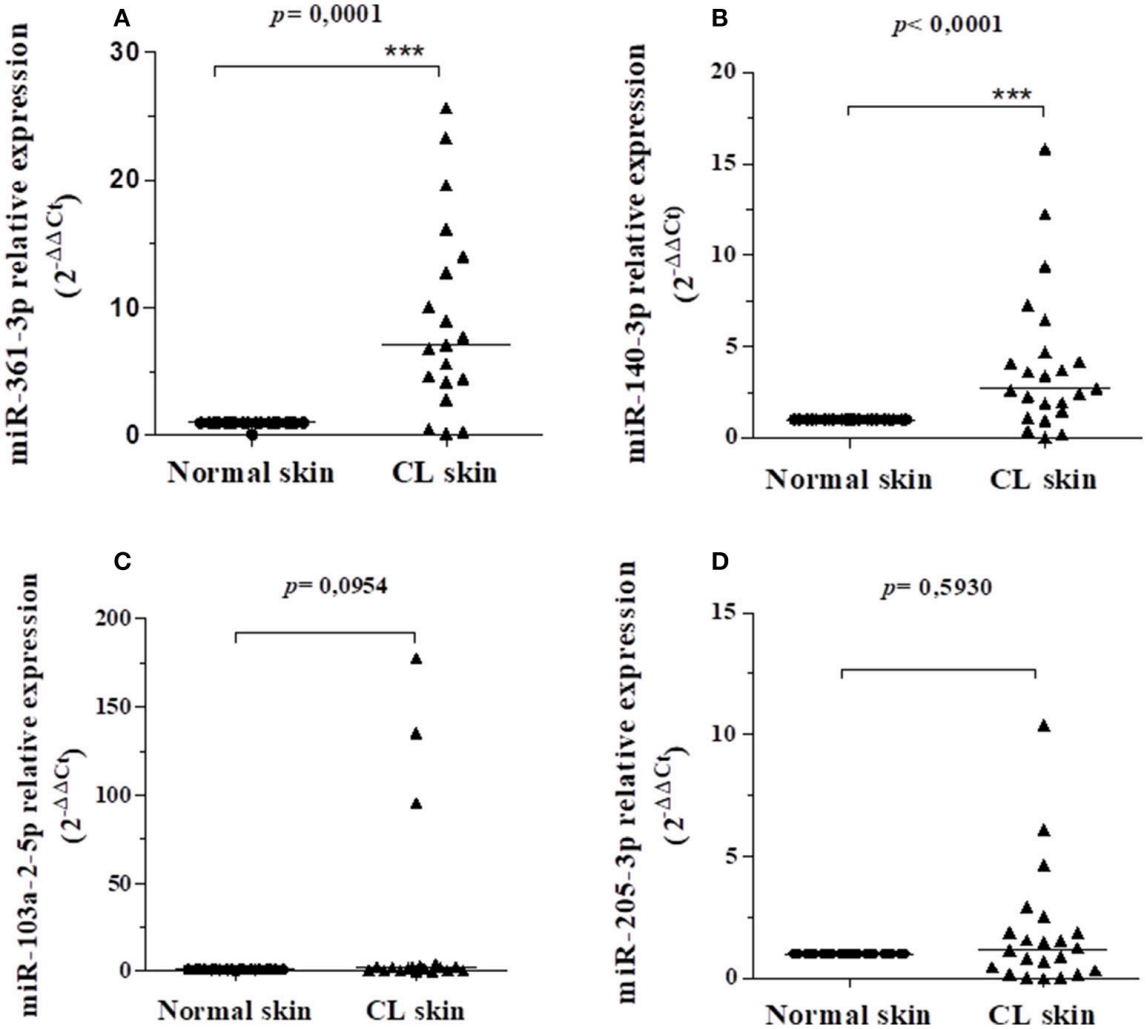
Relative gene expression of miR-361-3p **(A)**, miR-140-3p **(B)**, miR-103a-2-5p **(C)**, and miR-205-3p **(D)** between CL skin and normal skin samples from the same patient. All values were represented by the equation 2-ΔΔCt of the lesion in relation to the normal skin of each individual. The bars represent the median of the groups. Data were analyzed using the non-parametric Mann–Whitney test as implemented by the GraphPad Prism program. The miR-361-3p and miR-140-3p were significantly more expressed in LC lesions compared to normal skin samples (*p* = 0.0001 and *p* < 0.0001, respectively).

The miRNAs 361-3p, 103a-2-5p, 140-3p, 1265, 205-3p, 584-5p, 7161-3p, 3646, 1252-3p, 4313, 153-5p, 5011-5p, 6885-3p, and 4753-3p were evaluated in this study, according to their association with the differentially expressed target genes found in GSE55664 (Table 2). It is important to note that some of these miRNAs also target the *FLI1* gene that was previously associated to increased susceptibility to CL in families from Corte de Pedra (32). We compared the biopsies of CL lesions and normal skin from the same patient (*N* = 24) in order to determine if the pathogenesis of CL caused by *L. braziliensis* is accompanied by an altered expression of miRNAs that regulate genes expressed in CL lesions. Of the fourteen target miRNA evaluated, only four showed expression in the tissues: miR-361-3p,−103a-2-5p,−140-3p, and−205-3p. Interestingly, the miR-361-3p and miR-140-3p were significantly more expressed in the CL lesions when compared to normal skin samples (*p* = 0.0001 and *p* < 0.0001, respectively), as shown in Figures 1A,B. The miR-103a-2-5p and−205-3p although more expressed in lesions as compared to normal skin, did not reach statistical significance, as shown in Figures 1C,D.

**Table 2:** The miRNAs and their target genes.

**miRNA**	**# of access miRBase**	**Regulated genes**	**Mature miRNA sequence**
hsa-miR-361-3p	MIMAT0004682	TNF e GZMB	UCCCCCAGGUGUGAUUCUGAUUU
hsa-miR-103a-2-5p	MIMAT0009196	CXCL9, FLI1, GBP5, GNLY	AGCUUCUUUACAGUGCUGCCUUG
hsa-miR-140-3p	MIMAT0004597	NKG7	UACCACAGGGUAGAACCACGG
hsa-miR-1265	MIMAT0005918	CXCL10,GBP5,CXCL9	CAGGAUGUGGUCAAGUGUUGUU
hsa-miR-205-3p	MIMAT0009197	FLI1, CXCL9, IL1-β, IL6	GAUUUCAGUGGAGUGAAGUUC
hsa-miR-584-5p	MIMAT0003249	GBP5,CXCL9	UUAUGGUUUGCCUGGGACUGAG
hsa-miR-7161-3p	MIMAT0028233	GBP5,CXCL9,MMP1	UAGAUCUUUGACUCUGGCAGUCUCCAGG
hsa-miR-3646	MIMAT0018065	GZMA	AAAAUGAAAUGAGCCCAGCCCA
hsa-miR-1252-3p	MIMAT0026744	GZMA	CAAAUGAGCUUAAUUUCCUUUU
hsa-miR-4313	MIMAT0016865	GZMB	AGCCCCCUGGCCCCAAACCC
hsa-miR-153-5p	MIMAT0026480	CXCL9,CXCL10,GBP5,IDO1	UCAUUUUUGUGAUGUUGCAGCU
hsa-miR-5011-5p	MIMAT0021045	CXCL9,CXCL10,GBP5,IDO1	UAUAUAUACAGCCAUGCACUC
hsa-miR-6885-3p	MIMAT0027671	FLI1,GZMB,CXCL9	CUUUGCUUCCUGCUCCCCUAG
hsa-miR-4753-3p	MIMAT0019891	FLI1,MMP1,CCL8	UUCUCUUUCUUUAGCCUUGUGU

### Correlation of the miRNA expression with the clinical parameters of patients with LC (lesion size, treatment response and healing time)

**Figure 2:**
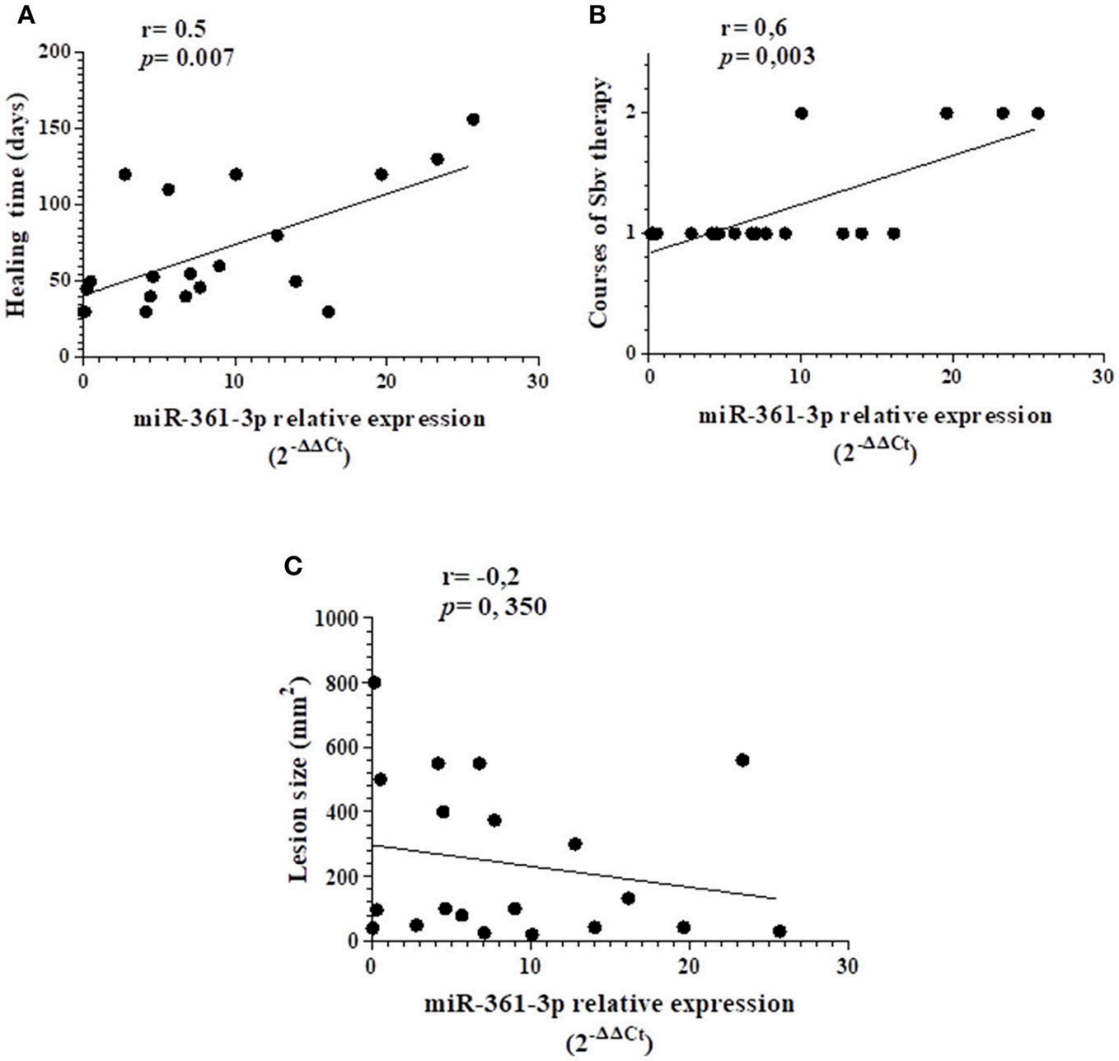
Spearman correlation analysis of the miR-361-3p in relation to healing time **(A)**, therapeutic failure **(B)**, and lesion area **(C)**. There was a positive correlation between this miRNA and the clinical parameters of therapeutic failure (*r* = 0.6, *p* = 0.003) and longer healing time (*r* = 0.5, *p* = 0.007).

We performed correlation analysis between the expression of the miRNAs and clinical parameters, lesion size, # of courses of antimony therapy and healing time. There was a positive correlation between the number of courses of antimonial pentavalent treatment and the expression of miR-361-3p, showing that the miRNA expression was higher in patients that fail to one therapy course of Sbv (*r* = 0.6, *p* = 0.003), Figure 2A. Moreover, we also found a positive correlation between the same miRNA and the healing time of CL (*r* = 0.5, *p* = 0.007), indicating that the higher individuals express the miR-361-3p, the longer is the time required to heal lesions, as shown in Figure 2B. The parameter of lesion size was not correlated with this miRNA (Figure 2C). There were no significant correlations between the other miRNAs and any of these clinical variables (data not shown).

### A ROC curve and survival analysis show that the expression of miR 361-3p can predict with good specificity and sensitivity the therapeutic failure and healing time in cL

**Figure 3:**
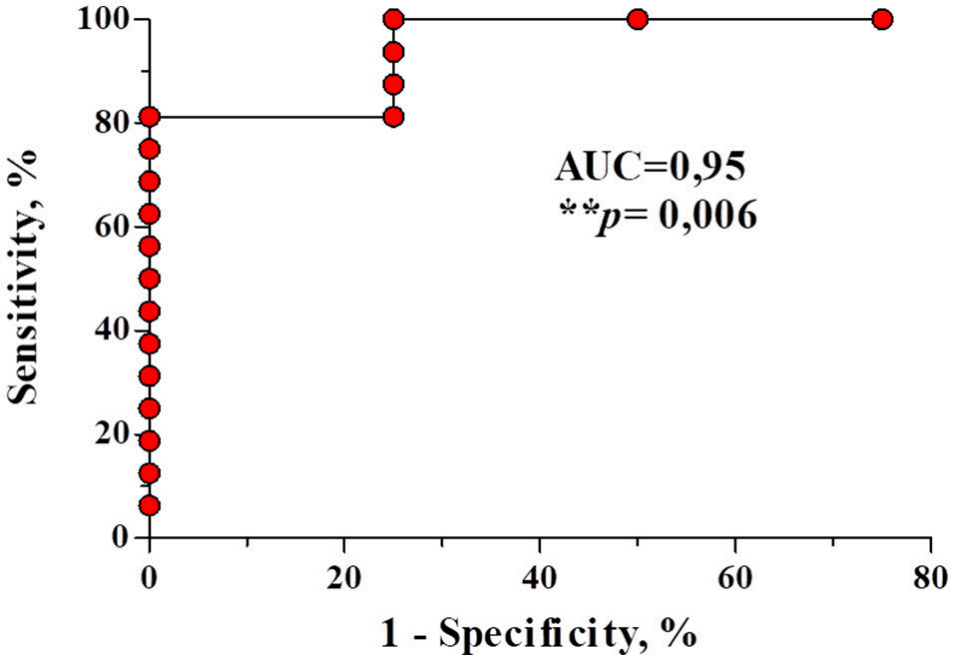
A ROC curve constructed using the relative expression values of miR-361-3p from refractory and responders patients after antimonial therapy. The miR-361-3p is able to identify with a sensitivity of 81.2% (IC = 54, 3–95, 9) and specificity of 100% (IC = 39, 7–100) patients who tend to fail initial treatment with Sbv.

**Figure 4:**
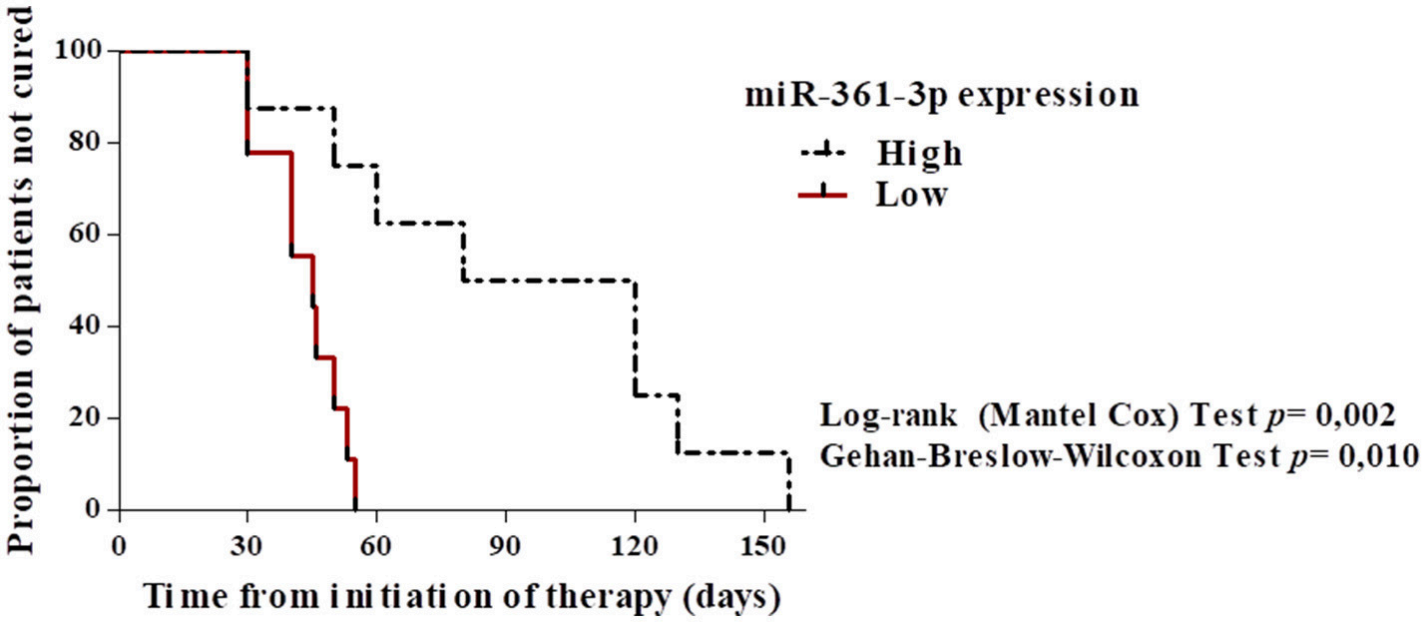
Kaplan–Meier survival analysis built considering “high” and “low” expression values of the miR-361-3p and “cure” as the outcome. Data showed the higher the expression of miR-361-3p, the longer the healing time of CL lesions (days).

To evaluate whether the expression of miR-361-3p can be used as a biomarker of therapeutic failure, we performed a ROC curve analysis, for which we have established a sensitivity of 81.2% (CI = 54.3–95.9) and specificity of 100% (CI = 39.7–100), using the cut off 9.5. This result shows that miR-361-3p expression values are able to correctly discriminate individuals who did not respond to treatment with one course of Sbv and are refractories (AUC = 0.95, *p* = 0.006), as shown on Figure 3. The same analysis was performed for the other miRNAs, but it was not significant (data not shown). In addition, we performed a Kaplan-Meier survival analysis considering cure as the outcome, with the objective of differentiating the healing time according to the expression values of miRNA 361-3p. The data revealed that the higher the expression of this miRNA, the longer the time required to heal lesions (*p* = 0.002 and *p* = 0.010), according to Figure 4.

## Discussion

Epigenetic modifications may provide an accessory source of fast-acting, reversible, and readily available phenotypic variation that can be directly shaped by both host and pathogen selection pressures (33, 34). In this sense, while molecular biologists will focus on identifying and characterizing circumstances where miRNAs are acting as gene regulatory switches, bioinformaticians will have to deal with the prospect that a substantial fraction of all animal mRNAs could have their level of expression influenced by miRNA regulation (35). The miRNAs has been widely described in various pathological conditions caused by parasites. In leishmaniasis, there is now a body of evidence showing that the host's miRNAs repertoire is affected upon Leishmania infection and that might interfere with immunity pathways (24, 25, 36, 37). Nevertheless, regarding LTA, the only documentation associating miRNAs with *L. braziliensis* infection was recently published by Nunes et al. (26). The present study shows that miR-361-3p and−140-3p were significantly more expressed in CL lesions as compared to the normal skin. Moreover, we document that the miR-361-3p expression was associated with failed to antimony therapy and consequently with a high healing time of the cutaneous ulcers.

As one of the aims of the present study was to evaluate if miRNA expression was a biomarker of severity of the disease and response to therapy we evaluated monthly the clinical evolution and response to therapy of 24 patients with CL. While there was no difference in the majority of the demographic and clinical manifestations in the two groups the mean age was lower in those who failed in comparison with the responders to antimony. Additionally the cure rate was higher than our more recent studies. The percentage of failure to therapy was lower than we have usually observed that similar to previous studies. We believe that the limited number of participants of the study was responsible for these differences (28–30).

The observation that the miR-361-3p and 140-3p were more expressed in CL lesions evidences that *L. braziliensis* parasites presence alters the expression of miRNAs in the tissue environment. Genes targeted by the miRNA miR-361-3p (*TNF, GZMB*) and miR-140-3p (*NKG7*) also had increased expression in the lesions of individuals infected by *L. braziliensis* (8). It is generally postulated that there is an inverse correlation between the miRNA expressions in relation to its target genes, that is, as miRNA expression increases in the microenvironment, the gene expression tends to go down. However, unlikely expected, we observed that both miR-361-3p and miR-140-3p were up regulated in the CL lesions. Thus, we hypothesized two possible explanations for this data: (1) This higher expression may be occurring as an attempt of these miRNAs to contain the also exacerbated expression of their target genes at the lesion site; or (2) Alternatively, these miRNAs could be inducing the expression of these genes at the promoter region. In the latter case, our results would be in agreement with the report of Zhang et al. (38), which showed that the miRNA let-7i associated to the RNA polymerase II in the cell nucleus, binds to the TATA box motif of interleukin-2 (IL-2) gene and elevates both IL-2 mRNA and protein in peripheral CD4+ T-lymphocytes of HIV-1 subjects. Likewise, other cellular miRNAs such as miR-138 and miR-92a also increase promoter activities by binding to TATA-box motifs (38). Additionally, further studies have demonstrated that several miRNAs can induce gene expression by binding to complementary sequences in the promoter (39, 40). All together, these findings indicate an additional regulatory mechanism for gene expression by miRNAs. Thus, we postulate that increased expression of miR-361-3p and miR-140-3p can up regulate its target genes in lesions of patients infected by *L. braziliensis*, collaborating to the worsening of tissue damage. However, we cannot ignore that these miRNAs could be up-regulated as a way to modulate other target genes in the lesions. As an example, the miR 361-3p also targets the Filaggrin-2 (*FLG2*) gene, associated with maintenance of the epidermal barrier function. To consubstantialize this hypothesis, previous transcriptome data (8) showed that GZMB and TNF were 50.9 and 4.51 times, respectively, more expressed in lesion skin as compared to normal skin, whereas the FC (fold change) for FLG2 were only 0,063 in the same comparison, placing it as one of the least expressed genes in the CL lesions.

Alternatively this miRNA may be implicated with a pathological response as its expression could be part of the cytotoxic mechanism mediated by the recruitment of NK and CD8+T cells in response to *L. braziliensis* infection. These cell types induce the pro-inflammatory cytokine TNF increasing cellular adhesion, necrosis and cytotoxicity (4, 5, 41). Moreover, previous data documented a positive correlation between the frequency of CD8+ T cell expressing granzyme B+ and lesion size (10). Granzyme B can indirectly promote inflammation through the activation of cytokines such as IL-18, IL-1β, and IL-1α (42–44). One probable mechanism of how cytotoxic cells mediate inflammation and tissue injury in CL is that, after degranulation of cytotoxic cells, granzyme B, and perforin are released into the extracellular space, inducing apoptosis of infected macrophages and bystander cells. In addition, extracellular granzyme B may indirectly contribute to inflammation by activating pro-inflammatory cytokines such as TNF, as well as degradation of ECM substrates, resulting in tissue injury (45). The search for correlations with clinical parameters was a strategy employed in this study in order to explore potentially relevant data in the context of CL pathogenesis. Thus, illness duration, response to treatment and healing time were parameters selected for correlations with the expression of detected miRNAs. Our results show, in a pioneering way, a direct correlation between the expression levels of a miRNA with both therapeutic failure and healing time in CL. Furthermore, additional analysis showed that the expression levels of miR-361-3p can predict with high sensitivity and specificity patients that will not respond to the treatment with one course of Sbv and are potential refractories. Likewise, survival analysis having the cure as the outcome showed that the higher the miR-361-3p expression, the longer the healing time of CL lesions. These results have an outstanding impact because it indicates that miR-361-3p could have a prognostic value in the evolution of CL as a biomarker of failure to therapy. The healing time of a CL ulcer is slow and a second course of Sbv or use of an alternative drug is only initiated after 90 days of the initiation of therapy. Our data indicate that a high expression of 361-3p before therapy may indicate a patients who have a high rate to failure to antimony therapy. In such case alternative drugs may be used rather than antimony. Moreover, our results open perspectives for new exploratory studies involving miRNAs, as well as functional studies that may aggregate new information useful for the development of new therapeutic targets in LTA.

## Author contributions

TL conducted molecular biology experiments and analyzed and interpreted data. JS supported the sample collection. EL assisted in the recruitment and segment of patients. EC enrolled the study participants and helped to analyze data and draft the manuscript. DZ participated in the study design and helped with quality control and data analysis. LC conceived the study and participated in its design, helped to analyze and interpret the data, and drafted the manuscript.

### Conflict of interest statement

The authors declare that the research was conducted in the absence of any commercial or financial relationships that could be construed as a potential conflict of interest.
